# Arabic handwritten alphabets, words and paragraphs per user (AHAWP) dataset

**DOI:** 10.1016/j.dib.2022.107947

**Published:** 2022-02-13

**Authors:** Majid Ali Khan

**Affiliations:** College of Computer Engineering and Science, Prince Mohammad Bin Fahd University, Khobar, Eastern Province, Saudi Arabia

**Keywords:** Handwritten Arabic alphabets, Handwritten Arabic words, Handwritten Arabic paragraphs, Writer identification, Arabic Text recognition

## Abstract

This article presents a handwritten Arabic alphabets, words and paragraphs dataset (AHAWP). The dataset contains 65 different Arabic alphabets (with variations on begin, end, middle and regular alphabets), 10 different Arabic words (that encompass all Arabic alphabets) and 3 different paragraphs. The dataset was collected anonymously from 82 different users. Each user was asked to write each alphabet and word 10 times. A userid uniquely but anonymously identifies the writer of each alphabet, word and paragraph. In total, the dataset consists of 53199 alphabet images, 8144 words images and 241 paragraphs images. This dataset can be used for multiple purposes. It can be used for optical handwriting recognition of alphabets and words. It can also be used for writer identification (or verification) of handwritten Arabic text. It is also possible to evaluate difference in writing styles of isolated alphabets as compared to the same alphabet written as part of the word or in paragraph by the same user using this dataset. The dataset is publicly available at https://data.mendeley.com/datasets/2h76672znt/1.

## Specifications Table

**Table:** 

Subject	Computer Science
Specific subject area	Image processing, Optical Handwritten Text Recognition, Writer Identification
Type of data	Image
How the data were acquired	Users completed the forms (based on fixed template) with their handwriting and these forms were then scanned
Data format	Raw
Description of data collection	Data was collected anonymously in a classroom setting. A “userid” was used to uniquely but anonymously identify the writer of each alphabet, word and paragraph. The forms were color scanned at 300 dpi and handwritten Arabic alphabets, words and paragraphs were then cropped from these scanned forms.
Data source location	College of Computer Engineering and Science, Prince Mohammad Bin Fahd University City/Town/Region: Khobar Country: Saudi Arabia Latitude and longitude for collected samples/data: 26.14544181406805, 50.091155268800044
Data accessibility	Repository name: Mendeley Data Data identification number: 10.17632/2h76672znt.1 Direct URL to data: https://data.mendeley.com/datasets/2h76672znt/1

## Value of the Data

• This data is useful for optical handwriting recognition of Arabic text [1,2]. It can also be used for writer identification/verification of handwritten Arabic text [3], [4], [5]. This dataset contains handwritten alphabets, words and paragraphs written by the same user which makes it possible to develop writer identification models trained on alphabets and evaluated on words or sentences [6]
• The data can be used by machine learning researchers/companies to develop models for recognizing handwritten text. It can also be used by researchers/companies to develop forensic models for identifying writer of certain handwritten text.
• The existing Arabic alphabet identification datasets (Hijja [1], AHCD [7] datasets) do not provide any user information. The existing writer identification of Arabic text datasets (IFN/ENIT [8], KHATT [9], QUWI [10] datasets) only provide handwritten words or paragraphs and do not contain alphabets. This dataset fills in this gap.
• The existing Arabic datasets cannot be used for writer identification based on Arabic alphabets or to evaluate differences in writing styles of isolated alphabets as compared to the same alphabet written as part of the word or paragraph by the same user. This dataset can be used for this purpose as it captures the user information for each alphabet, word and paragraph.

## 1 Data Description

The dataset contains 65 different Arabic alphabets (with variations on begin, middle, end and regular alphabets), 10 different Arabic words and 3 different paragraphs handwritten by 82 writers. Each writer was asked to write each alphabet and word 10 times. A userid uniquely but anonymously identifies the writer of each alphabet, word and paragraph. In total, the dataset consists of 53199 alphabet images, 8144 words images and 241 paragraphs images.

The writers had fluent Arabic speaking and writing background. They were not advised to use any specific type or color of writing instrument. This resulted in a dataset with several variations in type and color of writing instruments. A single writer had typically used the same type and color of writing instrument to write the alphabets, words and paragraphs.

**Fig. 1:**
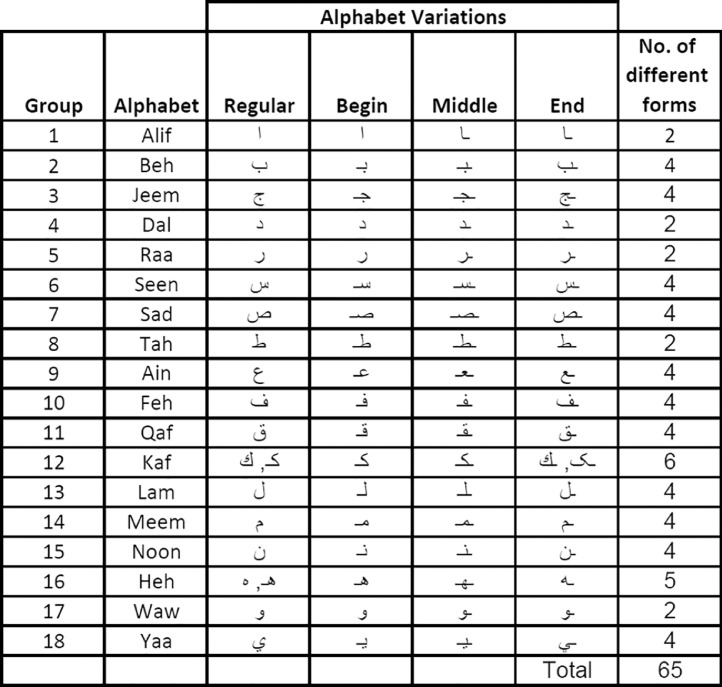
Arabic Alphabets with variations collected in the dataset.

Fig. 1 shows the set of Arabic alphabets collected in the dataset. Please note that a single alphabet is collected to represent a similarly styled group of alphabets.

**Fig. 2:**
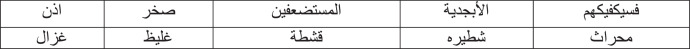
Arabic words collected in the dataset.

Dataset contains 10 different Arabic words (that encompass all Arabic alphabets) handwritten by 82 different users. Fig. 2 shows the set of Arabic words collected in the dataset.

**Fig. 3:**
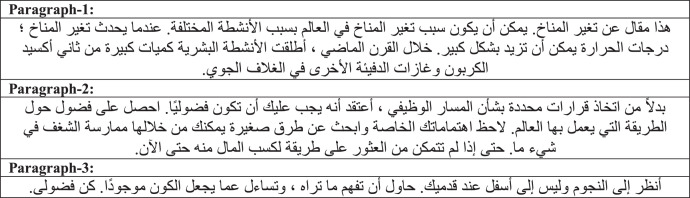
Arabic paragraphs collected in the dataset.

Dataset contains 3 different paragraphs handwritten by 82 different users. Fig. 3 shows the fixed text paragraphs collected in the dataset.

**Fig. 4:**
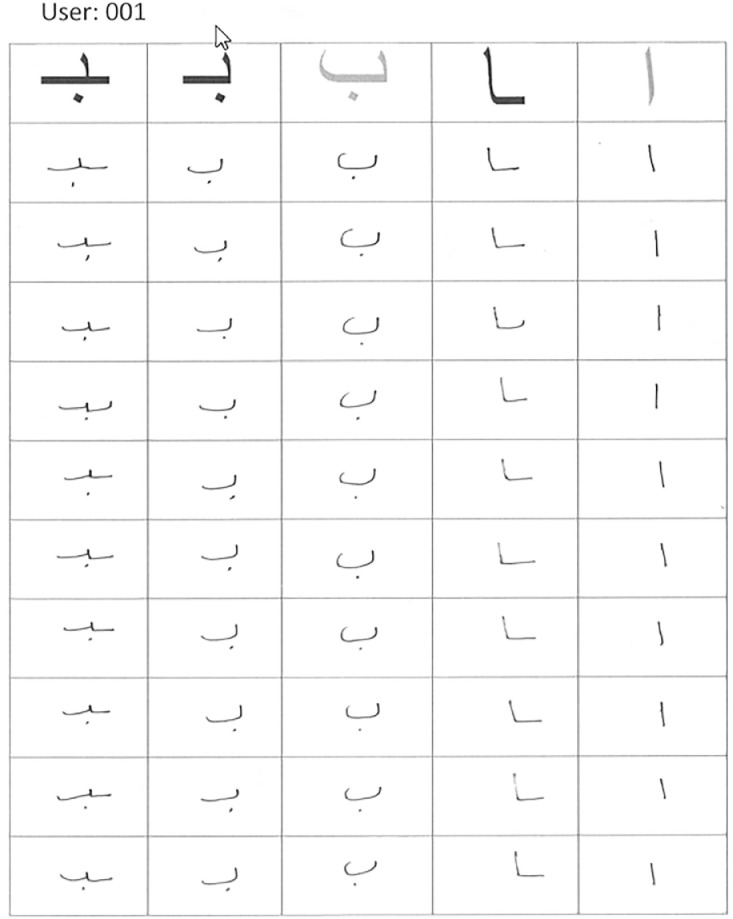
Arabic alphabets handwritten by a user.

**Fig. 5:**
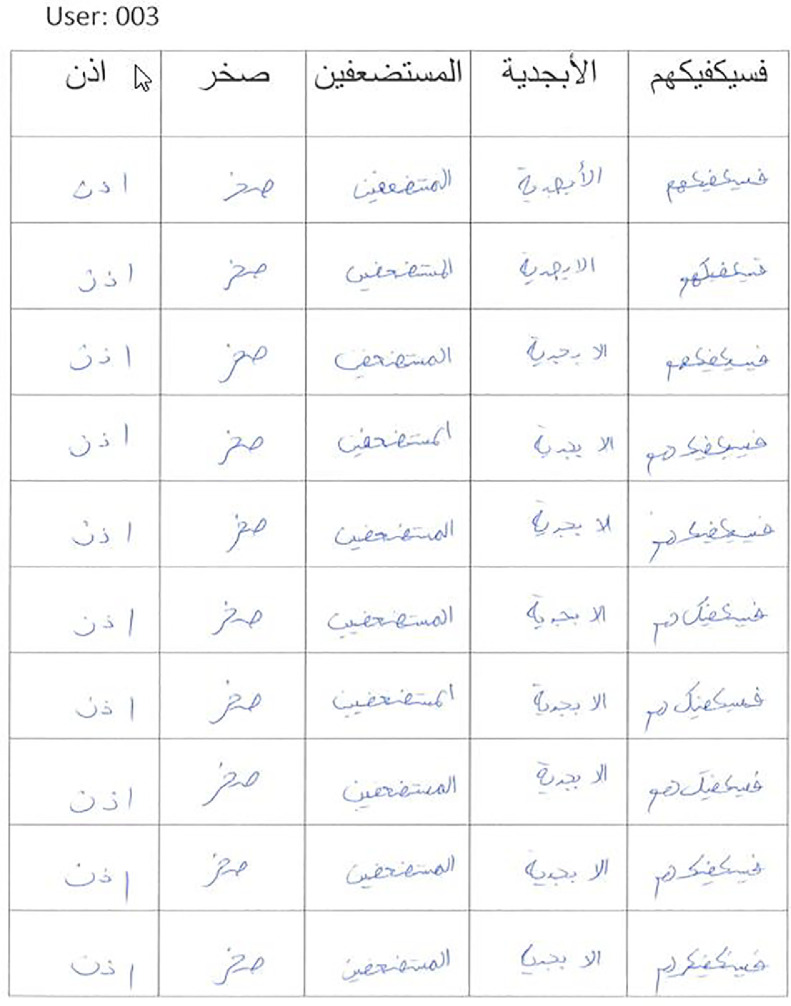
Arabic words handwritten by a user.

**Fig. 6:**
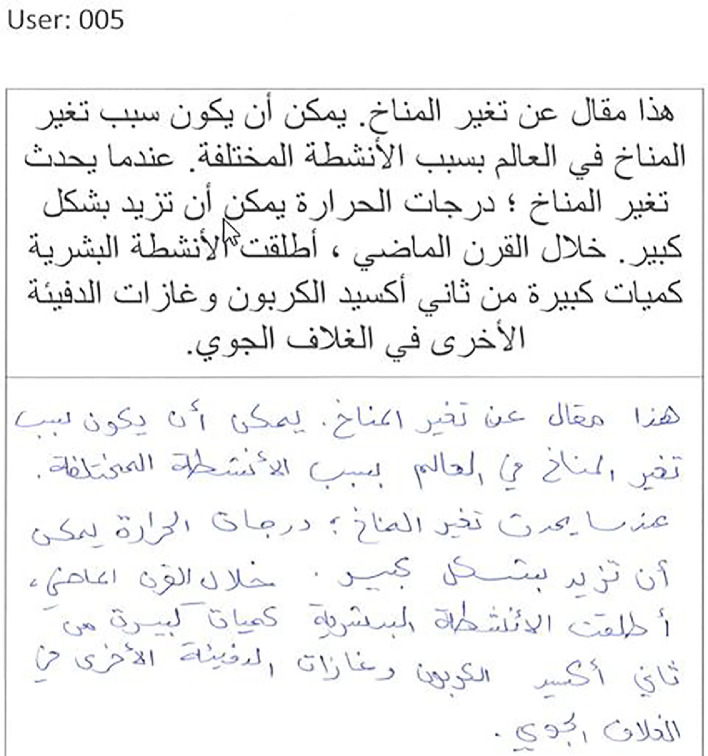
Arabic paragraph handwritten by a user.

Fig. 4 shows template of the form used to collect a sample of handwritten Arabic alphabets from a user (“user001”). Fig. 5 shows template of the form used to collect a sample of handwritten Arabic words from a user (“user003”). Fig. 6 shows template of the form used to collect a sample of handwritten Arabic fixed text paragraphs from a user (“user005”).

The handwritten forms from al the 82 users were color scanned at 300dpi resulting in an image resolution of 2480 × 3507 pixels. These scanned images are provided as raw data in the folder named “raw_dataset” in the public repository.

## 2 Experimental Design, Materials and Methods

The dataset from scanned images was extracted using Python scripts. The scripts are provided with the dataset in the public repository. Following is a brief description of the scripts:

• 1a_alphabet_extractor_per_alphabet.py: This script extracts alphabets from the scanned JPEG images (as shown in Fig. 4) and organizes them in a folder structure with one folder per alphabet containing that alphabet written by all the users. Each file name has the format “userid_alphabetName_variationName_index” where index increases sequentially for each extracted alphabet from a single page.
• 1a_alphabet_extractor_per_user.py: This script extracts alphabets from the scanned JPEG images (as shown in Fig. 4) and organizes them in a folder structure with one folder per user containing all the alphabets written by that user. Each file name has the format “userid_alphabetName_variationName_index” where index increases sequentially for each extracted alphabet from a single page.
• 2a_alphabets_pre_processing.py: This script is used to pre-process the extracted alphabets. It crops the alphabets from the center of image (excluding 20 pixels on each side). This was done to remove any borders surrounding the extracted alphabets. The surrounding whitespace around written alphabets was then removed. The resultant image was converted to grayscale and scaled to a height of 128 pixels (keeping the aspect ratio intact). Please note that keeping aspect ratio is important so that handwriting does not get distorted.
• 1w_word_extractor_per_user.py: This script extracts words from the scanned JPEG images (as shown in Fig. 5) and organizes them in a folder structure with one folder per user containing all the words written by that user. Each file name has the format “userid_wordName_index” where index increases sequentially for each extracted word from a single page.
• 2w_words_pre_processing.py: This script is used to pre-process the extracted words. It crops the words from the center of image (excluding 5 pixels on each side). This was done to remove any borders surrounding the extracted words. The surrounding whitespace around written words was then removed. The resultant image was converted to grayscale and scaled to a height of 128 pixels (keeping the aspect ratio intact). Please note that keeping aspect ratio is important so that handwriting does not get distorted.
• 1p_paragraph_extractor_per_user.py: This script extracts paragraphs from the scanned JPEG images (as shown in Fig. 6) and organizes them in a folder structure with one folder per user containing all the paragraphs written by that user. Each file name has the format “userid_paragraphNumber_index”.

## Ethics Statements

An informed consent was obtained from all participants that the collected dataset will be used for research purposes and can be made publicly available to research community. The consent is available on each data collection form page (in “raw_dataset” folder, under each data collection form). Moreover, the participant data has been fully anonymized.

Since the dataset was collected as part of classroom assignment of a course, it did not require any prior approval from the ethics committee.

## Declaration of Competing Interest

The authors declare that they have no known competing financial interests or personal relationships that could have appeared to influence the work reported in this paper.
